# Impact of gestational age on the management of acute appendicitis during pregnancy: A nationwide observational study

**DOI:** 10.1002/ijgo.15953

**Published:** 2024-10-23

**Authors:** Shunya Sugai, Yusuke Sasabuchi, Hideo Yasunaga, Shotaro Aso, Hiroki Matsui, Kiyohide Fushimi, Kosuke Yoshihara, Koji Nishijima

**Affiliations:** ^1^ Department of Obstetrics and Gynecology Niigata University Medical and Dental Hospital Niigata Japan; ^2^ Department of Real‐World Evidence, Graduate School of Medicine The University of Tokyo Tokyo Japan; ^3^ Department of Clinical Epidemiology and Health Economics, School of Public Health The University of Tokyo Tokyo Japan; ^4^ Department of Health Services Research, Graduate School of Medicine The University of Tokyo Tokyo Japan; ^5^ Department of Health Policy and Informatics Institute of Science Tokyo Graduate School of Medical and Dental Sciences Tokyo Japan

**Keywords:** abortion, acute appendicitis, antibiotics, appendectomy, conservative management, length of stay, premature birth

## Abstract

**Objective:**

To compare conservative management and appendectomy for acute appendicitis during pregnancy by trimester.

**Methods:**

This retrospective cohort study used data from a national inpatient database from July 2010 to March 2022. Pregnant women diagnosed with acute appendicitis were included. Multivariable analysis using generalized estimating equations was performed to compare outcomes between conservative management and appendectomy across trimesters. The main outcomes were preterm labor, preterm delivery, or abortion; antepartum hemorrhage; duration of hospitalization; and duration of antibiotic use.

**Results:**

A total of 3158 individuals from 632 acute‐care hospitals were eligible. The proportion of conservative management versus appendectomy by trimester were 507 (49.1%) versus 525 (50.9%) in the first, 690 (44.6%) versus 856 (55.4%) in the second, and 337 (58.1%) versus 243 (41.9%) in the third. In the second trimester, appendectomy was associated with a higher rate of preterm delivery, preterm labor, or abortion (odds ratio [OR], 2.91 [95% confidence interval (CI), 1.62–5.25]). Antepartum hemorrhage occurred more frequently for appendectomy in the first (OR, 2.12 [95% CI, 1.31–3.43]) and third (OR, 2.43 [95% CI, 1.79–3.31]) trimesters. Appendectomy was associated with a longer duration of hospitalization in the second (2.15 days; 95% CI, 1.14–3.17 days) and third (3.97 days; 95% CI, 2.22–5.71 days) trimesters. Antibiotic use duration was shorter for appendectomy in the first (−1.20 days [95% CI −1.51 to −0.90 days]) and second (−0.61 days [95% CI −0.90 to −0.32 days]) trimesters.

**Conclusions:**

Clinical outcomes of acute appendicitis during pregnancy vary by trimester. Considering the appendectomy risks, conservative management may be viable depending on the clinical context and trimester.

## Synopsis

Pregnancy‐specific adverse events were more common in the appendectomy group than in the conservative management group, and the outcomes varied by trimester.

## 1 INTRODUCTION

Acute appendicitis during pregnancy can lead to maternal complications, such as sepsis and peritonitis, as well as fetal complications, including death and preterm labor.^1^, ^2^, ^3^ Consequently, the management of acute appendicitis during pregnancy requires careful consideration of complications that can affect the mother and fetus. The primary management options for acute appendicitis are conservative management or surgical treatment such as appendectomy. Several studies have suggested that appendectomy is associated with lower risks of sepsis, miscarriage, and fetal death than conservative management,^4^, ^5^, ^6^ while other studies have indicated that conservative management and appendectomy have similar outcomes.^7^, ^8^, ^9^

Because changes occur in both the mother and fetus as pregnancy progresses, gestational age is a critical factor for the selection of management.^10^ However, previous large‐scale studies comparing conservative management and appendectomy have provided insufficient information regarding gestational age, and thus the relationship between gestational age and management remains unclear.^2^, ^6^, ^11^ In other words, the safety and efficacy of conservative management and appendectomy according to gestational age are not well established.^12^

We utilized a nationwide inpatient database to stratify acute appendicitis during pregnancy by trimester and compare inpatient outcomes between conservative management and appendectomy. The study enabled us to evaluate the differences in safety and efficacy of conservative management and appendectomy according to gestational age, with the aim of informing recommendations for clinical practice.

## 2 MATERIALS AND METHODS

This retrospective cohort study examined data from July 2010 to March 2022 extracted from the Diagnostic Procedure Combination (DPC) database, which acts as the nationwide repository for hospitalized patients receiving acute care in Japan. The database collects discharge summaries and administrative claims data for patients in >1500 acute‐care hospitals, representing approximately 90% of all tertiary‐care emergency hospitals in Japan.^13^, ^14^ While academic hospitals are obliged to provide data, the participation of community hospitals is voluntary. Ethical approval was obtained from the institutional review board of The University of Tokyo (approval number: 3501‐(5) on May 19, 2021) to ensure adherence to the ethical standards outlined in the Declaration of Helsinki. Given the anonymized and deidentified nature of the data, the requirement for informed consent was waived.

The database includes a variety of information for patients, including sex, age, height, weight, main diagnosis, admission‐precipitating diagnosis, most resource‐consuming diagnosis, second most resource‐consuming diagnosis, comorbidities present on admission, and complications arising after admission, documented using *International Classification of Diseases, Tenth Revision* (*ICD‐10*), codes and Japanese text.^13^ It also contains information on procedures using Japanese medical procedure codes, prescribed medications, ambulance use, admission to a teaching hospital, pregnancy status, gestational age, and dates of admission, discharge, procedures, and prescriptions.^13^ The reliability of the diagnostic data in the database has been confirmed, with the primary diagnosis accuracy having a sensitivity of 50% to 80% and a specificity of 96%.^15^ Recorded procedures also have high accuracy, with sensitivity and specificity both exceeding 90%.^15^

We identified pregnant women diagnosed with acute appendicitis using the following *ICD‐10* codes: K350, K351, K352, K353, K358, and K359. Complicated appendicitis comprised K350 or K352 (acute appendicitis with generalized peritonitis) and K351 or K353 (acute appendicitis with peritoneal abscess). Noncomplicated appendicitis comprised K358 or K359. K350, K351, and K359 are outdated codes. Individuals who underwent appendectomy during hospitalization were categorized into the appendectomy group, identified by the Japanese medical procedure codes K718 and K718‐2. K718 indicates appendectomy by laparotomy, while K718‐2 denotes appendectomy by laparoscopy. Individuals who did not undergo surgical treatment were categorized into the conservative management group. Based on gestational age at admission, the first trimester was defined as <14 weeks, the second trimester as ≥14 weeks but <28 weeks, and the third trimester as ≥28 weeks. We compared the outcomes for conservative management and appendectomy by trimester.

The following data were examined: age, body mass index, comorbidities, ambulance use, admission to a teaching hospital, and gestational age at admission. Comorbidities were identified using *ICD‐10* codes as follows: preexisting hypertension (I10, I15x, O100, O104, O109), diabetes (E10x, E11x, E12x, E13x, E14x, O24x), and multiple gestation (O30x). The variable of multiple gestation was used for exclusion during patient selection.

The main outcomes included preterm labor, preterm delivery, or abortion (O021, O03x, O068, O364, O60x), antepartum hemorrhage (O20x, O441, O45x, O469), duration of hospitalization, and duration of antibiotic use.

Continuous variables are presented as median and interquartile range, while categorical variables are presented as number and percentage. Comparisons of data between the conservative management group and appendectomy group were conducted using the Mann–Whitney *U* test for continuous variables and Fisher's exact test for categorical or binary data. In the analysis of outcomes, multivariable analysis with generalized estimating equations was used to adjust for individual‐level and hospital‐level factors. For binary outcomes, multivariable logistic regression was employed to calculate the odds ratio (ORs) and 95% confidence intervals (CIs). For continuous outcomes, multivariable linear regression was additionally used to calculate the *β* coefficient and 95% CI. In all regression analyses, the women were stratified by trimester to estimate the effect of appendectomy versus conservative management on outcomes. The statistical analyses were conducted using Stata/SE 17 (StataCorp) and R version 4.3.0 (R Foundation for Statistical Computing). All tests were two‐tailed, and the threshold for statistical significance was *P* < 0.05.

## 3 RESULTS

**FIGURE 1:**
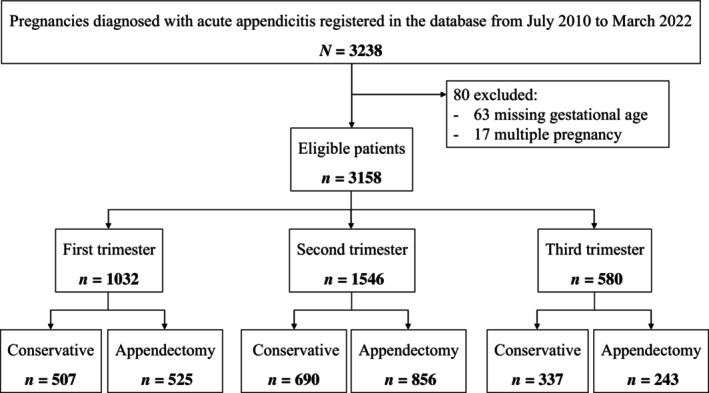
Flowchart of the study participants. In total, 3238 cases of acute appendicitis during pregnancy were identified in the database. From these, cases with unknown gestational age and multiple pregnancies were excluded. Finally, 3158 individuals met the eligibility criteria: 1032 (32.7%) in the first trimester, 1546 (49.0%) in the second trimester, and 580 (18.4%) in the third trimester.

The flowchart for patient selection is shown in Figure 1. During the study period, we identified 3238 pregnant women with acute appendicitis in the database. Of these, 3158 from 632 facilities were deemed eligible for the study. Among the eligible patients, 1032 (32.7%) were in the first trimester, 1546 (49.0%) in the second trimester, and 580 (18.4%) in the third trimester. The applications of conservative management versus appendectomy by trimester were 507 (49.1%) versus 525 (50.9%), 690 (44.6%) versus 856 (55.4%), and 337 (58.1%) versus 243 (41.9%), respectively. Among the appendectomy cases, the proportions of laparoscopic appendectomy by trimester were 188 (35.8%), 350 (40.9%), and 66 (27.2%), respectively. The median time to surgery after hospital admission across all trimesters was 1 day (interquartile range, 1–2 days).

**FIGURE 2:**
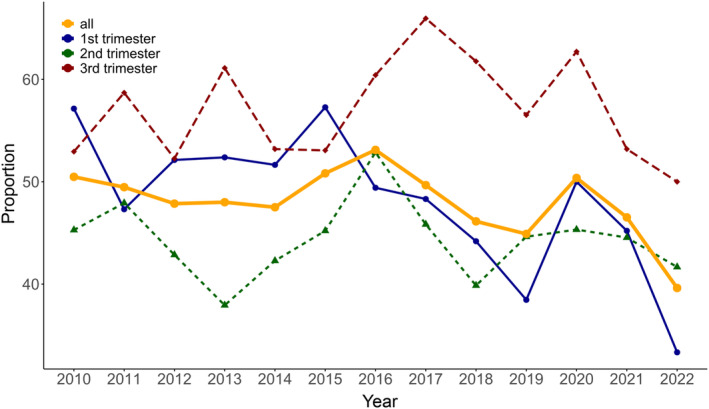
Annual trends in conservative management rate. The denominator includes both conservative management and appendectomy, while the numerator consists solely of conservative management. The proportion of conservative management decreased from 50.5% in 2010 to 39.6% in 2022, with a notable decrease from 57.1% to 33.3% in the first trimester.

Table 1 presents the baseline characteristics of the pregnant women diagnosed with acute appendicitis by trimester. Age and body mass index were similar in all groups. Ambulance use and admission to a teaching hospital tended to be higher in the appendectomy group. The proportion of complicated appendicitis cases was significantly higher in the appendectomy group in all trimesters. Figure 2 shows the annual trends in the proportion of conservative management. The annual proportion of conservative management across all trimesters decreased from 50.5% in 2010 to 39.6% in 2022. In the first trimester in particular, the annual proportion decreased from 57.1% to 33.3%.

**TABLE 1:** Baseline characteristics of the patients undergoing conservative management versus appendectomy.

Characteristics	First trimester			Second trimester			Third trimester		
	Conservative (*n* = 507)	Appendectomy (*n* = 525)	*P* value	Conservative (*n* = 690)	Appendectomy (*n* = 856)	*P* value	Conservative (*n* = 337)	Appendectomy (*n* = 243)	*P* value
Age (years)	29 (26–33)	30 (26–34)	0.072	30 (26–34)	31 (27–34)	0.168	31 (27–35)	32 (28–35)	0.323
Body mass index (kg/m^2^)			0.168			0.210			0.197
<18.5	105 (20.7)	88 (16.8)		69 (10.0)	87 (10.2)		7 (2.1)	10 (4.1)	
18.5–24.9	304 (60.0)	348 (66.3)		477 (69.1)	628 (73.4)		201 (59.6)	160 (65.8)	
25.0–29.9	45 (8.9)	40 (7.6)		81 (11.7)	85 (9.9)		95 (28.2)	53 (21.8)	
≥30.0	15 (3.0)	11 (2.1)		19 (2.8)	14 (1.6)		19 (5.6)	13 (5.3)	
Missing	38 (7.5)	38 (7.2)		44 (6.4)	42 (4.9)		15 (4.5)	7 (2.9)	
Gestational age (weeks)	9 (7–12)	10 (7–12)	0.831	19 (16–22)	19 (16–23)	0.432	32 (30–36)	31 (29–34)	0.004
Smoking	70 (13.8)	81 (15.4)	0.545	70 (10.1)	84 (9.8)	0.471	25 (7.4)	17 (7.0)	0.794
Ambulance use	89 (17.6)	110 (21.0)	0.180	147 (21.3)	232 (27.1)	0.009	89 (26.4)	92 (37.9)	0.004
Teaching hospital	458 (90.3)	503 (95.8)	<0.001	622 (90.1)	822 (96.0)	<0.001	288 (85.5)	225 (92.6)	0.008
Preexisting hypertension	1 (0.2)	2 (0.4)	1.000	3 (0.4)	4 (0.5)	1.000	1 (0.3)	1 (0.4)	1.000
Diabetes	2 (0.4)	3 (0.6)	1.000	13 (1.9)	12 (1.4)	0.544	13 (3.9)	10 (4.1)	1.000
Complicated appendicitis	42 (8.3)	105 (20.0)	<0.001	65 (9.4)	179 (20.9)	<0.001	53 (15.7)	77 (31.7)	<0.001

*Note*: Data are presented as median (interquartile range) or number (percentage).

Table 2 shows the comparisons of maternal outcomes between the appendectomy group and the conservative management group stratified by trimester. In the second trimester, the appendectomy group had a significantly higher frequency of preterm delivery, preterm labor, or abortion compared with the conservative management group. Antepartum hemorrhage was more frequent in the appendectomy group in both the first and second trimesters. The duration of hospital stay was significantly longer in the appendectomy group in the second and third trimesters. The duration of antibiotic use was significantly shorter in the appendectomy group in the first and second trimesters.

**TABLE 2:** Maternal outcomes of conservative management versus appendectomy.

Outcomes	First trimester			Second trimester			Third trimester		
	Conservative (*n* = 507)	Appendectomy (*n* = 525)	*P* value	Conservative (*n* = 690)	Appendectomy (*n* = 856)	*P* value	Conservative (*n* = 337)	Appendectomy (*n* = 243)	*P* value
Preterm delivery, preterm labor, or abortion	7 (1.4)	11 (2.1)	0.478	16 (2.3)	55 (6.4)	<0.001	38 (11.3)	36 (14.8)	0.210
Antepartum hemorrhage	43 (8.5)	93 (17.7)	<0.001	86 (12.5)	220 (25.7)	<0.001	3 (0.9)	4 (1.6)	0.460
Length of stay (days)	6 (4–8)	6 (5–8)	0.283	6 (4–8)	7 (5–10)	<0.001	7 (6–10)	11 (8–16)	<0.001
Length of antibiotic use (days)	4 (3–6)	3 (2–4)	<0.001	4 (3–6)	4 (2–6)	<0.001	5 (3–6)	5 (3–7)	0.089

*Note*: Data are presented as median (interquartile range) or number (percentage).

Table 3 presents the results of the logistic regression analysis with generalized estimating equations. In the second trimester, the appendectomy group exhibited significantly higher odds for preterm delivery, preterm labor, or abortion compared with the conservative management group. However, no significant differences were observed in the first and third trimesters. The appendectomy group had higher odds for antepartum hemorrhage compared with the conservative management group in both the first and second trimesters but not in the third trimester.

**TABLE 3:** Multivariable logistic regression analyses for outcomes.

Outcomes	First trimester		Second trimester		Third trimester	
	OR (95% CI)	*P* value	OR (95% CI)	*P* value	OR (95% CI)	*P* value
Preterm delivery, preterm labor, or abortion^a^	1.74 (0.67–4.50)	0.255	2.91 (1.62–5.25)	<0.001	1.35 (0.80–2.27)	0.258
Antepartum hemorrhage^b^	2.12 (1.31–3.43)	0.002	2.43 (1.79–3.31)	<0.001	1.55 (0.32–7.43)	0.583

*Note*: The reference group was the conservative management group.

Abbreviations: CI, confidence interval; OR, odds ratio.

^a^ Adjusted for complicated appendicitis in the first and second trimesters, and for complicated appendicitis, age, and body mass index in the third trimester.

^b^ Adjusted for complicated appendicitis, age, body mass index, and ambulance use in the first trimester, the same factors plus admission to a teaching hospital in the second trimester, and complicated appendicitis in the third trimester.

Table 4 shows the results of the multivariable linear regression analyses with generalized estimating equations. The length of hospital stay was significantly longer in the appendectomy group compared with the conservative management group in the second and third trimesters. Duration of antibiotic use was significantly shorter in the appendectomy group compared with the conservative management group during the first and second trimesters.

**TABLE 4:** Multivariable linear regression analyses for outcomes.

Outcomes	First trimester		Second trimester		Third trimester	
	*β* (95% CI)	*P* value	*β* (95% CI)	*P* value	*β* (95% CI)	*P* value
Length of stay (days)	0.24 (−0.30 to 0.78)	0.388	2.15 (1.14–3.17)	<0.001	3.97 (2.22–5.71)	<0.001
Length of antibiotic use (days)	−1.20 (−1.51 to −0.90)	<0.001	−0.61 (−0.90 to −0.32)	<0.001	0.49 (−0.43 to 1.41)	0.297

*Note*: The reference group was the conservative management group. Adjusted for complicated appendicitis, age, body mass index, ambulance use, and admission to a teaching hospital.

Abbreviation: CI, confidence interval.

## 4 DISCUSSION

The present study underscores the influence of gestational age on the management of acute appendicitis during pregnancy. There were two principal findings. First, individual characteristics and management preferences for acute appendicitis during pregnancy are delineated by trimester. Second, for acute appendicitis during pregnancy, appendectomy was associated with increased risks of preterm delivery, preterm labor, abortion, and antepartum hemorrhage, as well as prolonged hospitalization, compared with conservative management depending on gestational age.

In our study, conservative management was used in 48.0% of cases across all trimesters. By trimester, the proportions were 49.1% in the first, 44.6% in the second, and 55.4% in the third. Previous studies have indicated a wide range of proportions for conservative management, varying from 9% to 67%.^5^, ^7^, ^8^, ^9^, ^16^ In guidelines from the United States and Europe, appendectomy is generally recommended for acute appendicitis during pregnancy.^10^, ^12^ Thus, the persistence of conservative management in a certain proportion of cases may arise from evidence for its efficacy in nonpregnant patients with acute appendicitis. Several studies have demonstrated potential benefits for conservative management of acute appendicitis in nonpregnant patients, as supported by randomized controlled trials and meta‐analyses.^17^, ^18^, ^19^, ^20^ Considering the adverse effects of surgery on the mother and fetus, conservative management may be preferable based on the available evidence in nonpregnant women with acute appendicitis.

In the present study, conservative management was more frequently chosen than appendectomy in the first and third trimesters than in the second trimester. In the first trimester, there may be a reluctance for surgery selection to minimize the risk of miscarriage.^10^ In the third trimester, the reluctance toward surgery may stem from the risks of fetal loss or preterm labor, the anatomical challenges posed by an enlarged uterus, and a preference for laparoscopic surgery to be limited to the second trimester.^2^, ^10^, ^21^, ^22^

The appendectomy group exhibited a higher incidence of antepartum hemorrhage in the first trimester than the conservative management group. In the second trimester, the appendectomy group had a higher probability of preterm delivery, preterm labor, or abortion, as well as an increased incidence of antepartum hemorrhage. In our cohort, we hypothesized that the higher incidence of complicated appendicitis in the appendectomy group was influential. However, after adjustment for this factor, there was no change in the outcomes. In a previous study that investigated similar outcomes to the present study, negative outcomes were observed when comparing the appendectomy group with the conservative management group,^11^ which is contrary to our findings. When comparing the previous study with the present study, significant differences are noted in the age demographics of the patients and the proportions of patients in the conservative management group versus the appendectomy group. Variations in healthcare conditions across different regions or countries may also have influenced the outcomes. However, a direct comparison of the previous study with the present study is difficult, because the previous study did not provide information on gestational age.

For nonpregnant women with acute appendicitis, appendectomy was reported to result in a shorter hospital stay.^17^, ^19^ In the present study, the duration of hospitalization was longer for the appendectomy group compared with the conservative management group in the second and third trimesters. The extended length of hospital stay for the appendectomy group is believed to be associated with postoperative pain, the risk of preterm labor, and concerns about fetal well‐being.^8^

Explaining the differences in outcomes across the trimesters is challenging. Specifically, it is difficult to disentangle and differentiate the risks associated with anesthesia, surgical techniques, and the nature and severity of the conditions that necessitate surgery.

In recent years, appendectomy has been regarded as the first choice for management of acute appendicitis during pregnancy.^12^ Although a few reports have suggested the effectiveness of conservative management,^7^, ^8^, ^9^ the associated investigations were small‐scale studies. The findings from the present large‐scale study indicate that conservative management may be a viable treatment option.

This study has several limitations. First, we utilized *ICD‐10* codes to identify cases of acute appendicitis in pregnancy. Although the DPC database is subject to rigorous checks of medical records, validation studies have not specifically focused on the population with acute appendicitis during pregnancy. Second, acute appendicitis is diagnosed by the attending physicians, and thus the diagnostic methods are presumed to vary among the cases. Moreover, the database does not contain data for pathological findings. The absence of this information may affect the applicability of our results to different patient populations. Third, the database lacks detailed clinical information such as symptoms, vital signs, and laboratory data. These factors could influence patient outcomes and may introduce bias into our study results. Fourth, owing to the limited number of outcomes, it was not feasible to incorporate a sufficient number of confounding factors in the logistic regression analysis. Fifth, we were unable to perform an external validation due to the lack of an independent data set. This limits the generalizability of our findings. Finally, the database does not contain data on postdischarge outcomes. Therefore, long‐term follow‐up data remain unavailable. Future studies should aim to include external validation and consider variables such as severity classification, comorbidities, and clinical status to enhance the robustness and applicability of the results.

Despite these limitations, the study possesses several strengths. It provides a comprehensive data set for acute appendicitis during pregnancy, with data collected from 632 facilities across Japan. Furthermore, we successfully collected data for a critical factor related to management during pregnancy, namely the trimester, which has not been accessible in previous studies utilizing large databases.^2^, ^6^, ^11^ Consequently, it became feasible to compare the clinical aspects of conservative management and appendectomy by trimester. Furthermore, through multivariable regression with generalized estimating equations, we were able to adjust for both individual‐level and hospital‐level factors.

## 5 CONCLUSION

The present findings highlight the variation in clinical characteristics of acute appendicitis during pregnancy across the trimesters. Given the potential for adverse outcomes associated with appendectomy, conservative management may be considered a viable option, taking into account the clinical context, including the trimester.

## AUTHOR CONTRIBUTIONS

All authors contributed to the study conception and design. Material preparation, data collection, and data analysis were performed by SS, YS, and SA. The first draft of the manuscript was written by SS. All authors critically revised the article and approved the final version.

## FUNDING INFORMATION

This work was supported by the Ministry of Health, Labour and Welfare, Japan (grant numbers 23AA2003 and 22AA2003).

## CONFLICT OF INTEREST STATEMENT

The authors report no conflict of interest.

## Data Availability

The data used in the study will not be made available since the data sets analyzed are not publicly available because of contracts with the hospitals providing data for the database.
